# Supplementary material for: Coronary Artery Disease in Patients Hospitalized for Peripheral Artery Disease: A Nationwide Analysis of 1.8 Million Patients
Source: Diagnostics (Basel). 2023 Mar 18;13(6):1163. doi: 10.3390/diagnostics13061163 (PMC10047185; doi:10.3390/diagnostics13061163)
Supplement: Supplementary file 1 [file diagnostics-13-01163-s001.zip › diagnostics-2257012-supplementary.pdf]

Table S1: Co-prevalence of peripheral artery disease (PAD) and coronary artery disease (CAD). Font. = Fontaine stage; Fontaine IVu = Fontaine IV with ulcers; Fontaine IVg = Fontaine IV with gangrene; CAD 0-3 = number of vessels with significant stenoses. Data are percentages.

**PAD**	**CAD**	**2009**	**2010**	**2011**	**2012**	**2013**	**2014**	**2015**	**2016**	**2017**	**2018**	**Mean**	**Change 2009-2018**
Font. IIb	CAD 0	1.5	1.4	1.4	1.5	1.6	1.3	1.4	1.4	1.3	1.2	1.4	-16.9
	CAD 1	2.8	2.7	2.9	3.1	3.1	3.3	3.6	3.5	3.8	3.8	3.3	+37.0
	CAD 2	3.6	3.8	3.7	3.8	3.8	3.8	4.3	4.4	4.5	4.4	4.0	+22.3
	CAD 3	6.8	7.0	7.0	7.3	7.9	8.0	8.6	9.2	9.2	9.1	8.0	+33.1
Font. III	CAD 0	1.5	1.5	1.4	1.6	1.5	1.4	1.3	1.5	1.3	1.5	1.4	-2.3
	CAD 1	2.7	2.7	3.2	3.4	3.2	3.5	3.9	4.0	3.8	4.0	3.4	+51.5
	CAD 2	3.8	4.1	3.9	4.0	4.2	4.5	4.6	4.9	5.0	4.8	4.4	+27.2
	CAD 3	7.9	8.1	8.5	8.8	9.2	9.6	9.5	10.8	10.8	10.8	9.4	+36.8
Font. IVu	CAD 0	1.6	1.5	1.4	1.4	1.3	1.3	1.2	1.4	1.2	1.1	1.3	-28.7
	CAD 1	2.1	2.1	2.3	2.6	2.9	3.1	3.3	3.7	3.8	4.0	3.1	+90.5
	CAD 2	3.1	3.4	3.3	3.4	3.7	3.9	4.1	4.5	4.4	4.9	3.9	+57.4
	CAD 3	7.1	7.8	7.9	8.3	8.9	9.7	10.4	11.1	11.0	11.5	9.6	+62.8
Font. IVg	CAD 0	1.5	1.4	1.3	1.3	1.3	1.1	1.1	1.2	1.2	1.0	1.2	-33.3
	CAD 1	2.2	2.1	2.4	2.7	2.9	3.0	3.5	3.7	3.9	3.9	3.0	+78.5
	CAD 2	3.1	3.4	3.4	3.8	4.0	4.0	4.4	4.7	4.9	4.8	4.0	+55.8
	CAD 3	7.5	8.0	8.8	9.5	10.2	11.4	11.5	12.3	12.5	12.9	10.5	+70.9
Total	CAD 0	1.5	1.4	1.3	1.4	1.5	1.3	1.3	1.4	1.3	1.2	1.4	-20.8
	CAD 1	2.5	2.5	2.7	2.9	3.0	3.2	3.6	3.7	3.8	3.9	3.2	+54.3
	CAD 2	3.4	3.7	3.6	3.8	3.9	4.0	4.3	4.5	4.6	4.6	4.1	+35.0
	CAD 3	7.2	7.5	7.8	8.2	8.8	9.2	9.7	10.4	10.4	10.6	9.0	+47.3
